# Relationship Between Perceived Risks of Using mHealth Applications and the Intention to Use Them Among Older Adults in the Netherlands: Cross-sectional Study

**DOI:** 10.2196/26845

**Published:** 2021-08-30

**Authors:** Nicky Sabine Klaver, Joris van de Klundert, Roy Johannes Gerardus Maria van den Broek, Marjan Askari

**Affiliations:** 1 Erasmus School of Health Policy & Management Erasmus University Rotterdam Netherlands; 2 Prince Mohammad Bin Salman College of Business & Entrepreneurship King Abdullah Economic City Saudi Arabia

**Keywords:** mHealth, older adults, perceived risks, intention to use, adoption, covid-19, digital health

## Abstract

**Background:**

Considering the increasing demand for health services by older people and the ongoing COVID-19 pandemic, digital health is commonly viewed to offer a pathway to provide safe and affordable health services for older adults, thus enabling self-management of their health while health care systems are struggling. However, several factors cause older people to be particularly reluctant to adopt digital health technologies such as mobile health (mHealth) tools. In addition to previously studied technology acceptance factors, those related to perceived risks of mHealth use (eg, leakage of sensitive information or receiving incorrect health recommendations) may further diminish mHealth adoption by older adults.

**Objective:**

The aim of this study was to explore the relationship between perceived risks of using mHealth applications and the intention to use these applications among older adults.

**Methods:**

We designed a cross-sectional study wherein a questionnaire was used to collect data from participants aged 65 years and older in the Netherlands. Perceived risk was divided into four constructs: privacy risk, performance risk, legal concern, and trust. Linear regression analyses were performed to determine the associations between these perceived risk constructs and the intention to use mHealth applications.

**Results:**

Linear regression per perceived risk factor showed that each of the four constructs is significantly associated with the intention to use mobile medical applications among older adults (adjusted for age, sex, education, and health status). Performance risk (*β*=–.266; *P*=<.001), legal concern (*β*=–.125; *P*=.007), and privacy risk (*β*=–.100; *P*=.03) were found to be negatively correlated to intention to use mHealth applications, whereas trust (*β*=.352; *P*=<.001) was found to be positively correlated to the intention to use mHealth applications.

**Conclusions:**

Performance risk, legal concern, and privacy risk as perceived by older adults may substantially and significantly decrease their intention to use mHealth applications. Trust may significantly and positively affect this intention. Health care professionals, designers of mHealth applications, and policy makers can use these findings to diminish performance risks, and tailor campaigns and applications to address legal and privacy concerns and promote mHealth uptake and health care access for older adults, especially during the COVID-19 pandemic.

## Introduction

Due to the shift in age distribution, an increasingly larger proportion of the world population will consist of adults over 65 years of age [1-3]. As we grow older, our demand for health care increases [4,5]. The global increase in the proportion of older adults is therefore expected to significantly increase global health care usage and costs [6]. To keep the health care system sustainable in the future, governments and societies are considering technology as a promising solution for health service delivery innovation and for service expansion without increasing human resource capacity [7-9].

Moreover, the COVID-19 outbreak has had extensive consequences for the provision of health care. This holds especially for older patients whose health care usage is likely to be higher while the pandemic makes it more critical for them to stay home, as age significantly determines the clinical features and prognosis of COVID-19 [10-12]. Through their rapid uptake, mobile health (mHealth) technologies enable health care without the need for face-to-face contact [10,13,14]. mHealth includes health-supporting applications on wireless devices such as tablets or smartphones [15-17]. These applications can assist independently living older adults, for instance, by monitoring clinical signs, collecting health information, or promoting a healthy lifestyle [18-21]. mHealth has shown to be able to improve care, self-management, and self-efficacy, as well as promote better behavior and medication adherence of older adults [14,19,22,23]. Unfortunately, however, older adults are less likely to adopt new technologies such as mHealth [24-28]. Before the pandemic, almost 50% of Dutch older adults had no intention to use mHealth applications [29].

Older people are deserving of special attention when it comes to technology adoption because of their different attitude toward technology [30,31]. For instance, they are more likely to perceive risks in the adoption of new technologies [32,33]. Potential risks, such as sensitive information leakage or incorrect health recommendations, may keep older people from using mHealth applications even during the COVID-19 pandemic when they are deemed especially beneficial. Earlier studies have shown that perception of risks are important barriers for mHealth acceptance and adoption [34-39]. Nevertheless, few studies have addressed the risks perceived by older people in relation to overall mHealth adoption or their intention to use such applications, which predominantly determines their adoption [40,41].

Deng et al [35] found that the intention to adopt mHealth is influenced by the perceived risks of using it among the general population. Perceived risks are one’s perception of uncertainty in the use of mHealth and include trust, performance risk, legal concern, and privacy risk. However, their study included very few respondents—those aged 65 years and older. Moreover, they focused on the hospital context where the use of mHealth applications is often limited to web-based consultation with a practitioner [35]. As a result, aging people who are not hospitalized remain an under-researched population concerning the perceived risks of mHealth adoption. Advancing the understanding of mHealth adoption among independently living older adults, therefore, has general importance because of their health care utilization and is especially relevant during the COVID-19 pandemic.

This study focuses on the perceived risks of mHealth adoption among independently living older adults. More specifically, the research aim is to determine the relationship between the risks of using mHealth applications as perceived by independently living older adults and their intention to use these applications. We set out to assess the validity and significance of perceived risks factors determining the intention to use the medical applications in a quantitative study involving a large sample of independently living older adults in the Netherlands.

## Methods

### Overview

The technology acceptance model (TAM) was developed to analyze the usage behavior of information technologies in organizational contexts [42]. TAM suggests that actual usage behavior is driven by the behavioral intention to use a system, as is also the case for the subsequent unified theory of acceptance and use of technology (UTAUT) model [43], and this has been empirically confirmed for mHealth usage among older adults [40,41,43]. Therefore, in this study, we selected behavioral intention to use mHealth applications from TAM as an outcome of interest. Building on the work of Askari et al [29], the statements operationalizing intention to use are taken from Venkatesh and Davis [44] and translated into Dutch, adding one new statement to account for linguistic differences. 

### Perceived Risk

Perceived risk, defined by Deng et al [35] as “one’s perception of uncertainty in the use of mHealth services and its severity in terms of consequences,” is measured with four constructs: privacy risk, performance risk, legal concern, and trust. In a 2018 empirical study, these authors identified an association between privacy risk, performance risk, legal concern, and trust with the intention to use mHealth in the general Chinese population. In this study, we adopted the statements, operationalizing these constructs from Deng et al [35] and translated them into Dutch to fit the context of independently living Dutch older adults. The corresponding English-language statements used to measure these constructs, as well as the questions to measure intention to use, are presented in Multimedia Appendix 1 [35,44]. Below, we explain the four different constructs that together capture perceived risk.

#### Privacy Risk

In this study, *privacy risk* refers to the extent to which an individual believes personal information abuse may occur because of mHealth application usage [35]. Previous studies on older adults have identified privacy concerns as a barrier to adopt health care technologies [45-47]. In our research context, we hypothesized that privacy concerns of older adults are negatively associated with their behavioral intention to use mHealth. We expect that older adults seek to have control over their lives as much as possible since perceived control is identified as an important factor in the well-being of aging people [48]. Sharing sensitive information over the internet may be perceived to diminish perceived control. Furthermore, older adults may be less familiar with technologies and, therefore, insecure about future destinations of personal information shared through an mHealth application.

#### Performance Risk

*Performance risk* is defined as the extent to which an individual doubts the capability of mHealth applications to realize desired outcomes [35]. Many older adults do not feel that they are able to use smartphones or tablets properly [30,49]. This could make them question the suitability of mHealth applications to manage their health. Moreover, many older people are skeptical that technology will replace health care professionals [45]. This also suggests they may question the quality and usability of health care technologies in comparison to traditional healthcare services. Therefore, we hypothesized that older adults are less likely to have a behavioral intention to use mHealth applications when perceiving performance risks.

#### Legal Concern

*Legal concern* refers to an individual’s worries regarding inappropriate law enforcement for mHealth applications [35]. For instance, older adults may prefer mHealth applications provided by third parties rather than by health care providers they visit, to prevent personal data to be illegally combined with clinical data. As our study targets mHealth applications to be used from home, the data protection is not covered by legislation applying to inpatient settings. Therefore, legal concern is hypothesized to be negatively associated with the intention to use mHealth applications. Our hypothesis is further corroborated by the lack of clarity about which laws cover mHealth disputes [50].

#### Trust

Finally, *trust* is defined as the perceived credibility of an mHealth application and the people behind it [35]. Older adults are less likely to trust assistive health care technologies [45]. Previous studies on the general population reveal a positive association between trust and the intention to use mHealth technologies [35,51-53]. We hypothesized the same association to hold for older adults.

### Study Design and Data Collection

A cross-sectional study was designed for this research, in which older adults over the age of 65 years were approached from February through June 2020. We developed a questionnaire and administered it digitally during the initial months of the COVID-19 pandemic. Data were collected by four data assistants in cooperation with different organizations, such as living facilities, senior citizen associations, and health service provider organizations, and via different web-based channels and mailing lists. The data has mainly been collected in the regions of Noord-Brabant, Utrecht, and Zuid-Holland. We have not been able to keep track of the number of recipients of the distributed web-based questionnaire as the cooperation organizations reached out to their clients and members for us. The number of completed questionnaires is reported in the Results section. The reporting of the web-based questionnaire follows the Checklist for Reporting Results of Internet E-Surveys (CHERRIES) checklist [54] and is presented in Multimedia Appendix 2.

The inclusion criteria for participants were as follows:

- The participant is 65 years of age or older
- The participant does not have cognitive impairments
- The participant lives independently

All respondents were asked to sign an informed consent form before participation. Thereafter, they could answer the questionnaire anonymously. The purpose of the project and information about the questionnaire, data management, and privacy of the participant were provided at the start of the questionnaire. Assistance and explanations were provided to participants who needed help filling out the questionnaire via telephone or email when requested. Data assistants entered the completed questionnaires into an SPSS database (IBM Corp) and pseudonymized the data to ensure anonymity.

### Statistical Analyses

#### Privacy Constructs and the Dependent Variable

The constructs privacy risk, performance risk, legal concern, and trust were adopted from a validated instrument and designed on a 5-point Likert scale, ranging from 1 = “strongly disagree” to 5 = “strongly agree” [35]. Per perceived risk construct, a score was computed by calculating the average score of all the related statements of that construct. These average scores acted as the independent variables in the analysis. The dependent variable *intention to use mHealth* was similarly calculated [55]. Participants with one or more missing values at the intention to use mHealth statements were deleted from further analysis. To test the internal reliability and validate the reliability of the statements for Dutch older adults, we calculated the Cronbach *α* for each construct. A construct was considered as reliable if Cronbach *α* was greater than .70 [56,57]. As an additional reliability test, a correlation matrix was calculated to test if the perceived risks factors were independent. Interdependent factors were not included together in the subsequent regression analysis to avoid multicollinearity [58].

#### Univariate and Multivariate Linear Regression Analyses

The calculated perceived risk factor scores served as an input for the univariate and multivariate linear regression analyses to examine the relationship between (each of) the perceived risk factors as independent variables and intention to use mHealth as the dependent variable. Univariate linear regression analyses were used to summarize the linear relationship between each perceived risk construct and intention to use mHealth, without controlling. Standardized coefficients with 95% CIs and *P* values were reported. Multivariate linear regression analyses were performed to calculate the unstandardized and standardized coefficients with 95% CIs and *P* values, to evaluate the relationship between each perceived risk construct and intention to use mHealth while controlling for age, sex, education, and health status. The rationale for choosing these control variables is explained below.

#### Control Variables

We included the following control variables: age, sex, education level, and health status. A correlation matrix was developed to test whether the control variables were independent [58]. The variables *sex* and *education level* were recoded into dummy variables as these variables are categorical variables. *Age* has been reported to be negatively related to intention to use technology [25,59]. Moreover, the risk of user errors increases with age due to an increase in physical and perceptual difficulties [60]. *Sex* was included as a control variable because previous studies on older adults determined that men are more likely to use technologies than women [27,59,61]. Women are generally more concerned than men [62]. Moreover, *education level* was included as a control variable, as more highly educated people are more likely to use mHealth technologies [35,59,63]. Finally, we included *health status* as a control variable since older adults may perceive more benefit from using mHealth as their health status would be relatively worse [63]. On the other hand, older adults with poorer health status may consider themselves more vulnerable and, therefore, more sensitive to perceived risks and mistrust [64].

### Validity and Reliability

Internal validity benefits from using validated instruments [35,42]. The questionnaire was validated by 5 experts (3 eHealth experts, 1 geriatric nurse, and 1 geriatrician). The usability and technical functionality of the web-based questionnaire was tested by 3 data assistants. The questionnaire is available on request. The database was checked for completeness and input errors by comparing a sample of paper questionnaires with the corresponding database information. To increase external validity, data collection took place in several different geographical locations within the Netherlands. Finally, Cronbach *α* was calculated for each factor to test reliability.

The study was approved by the Medical Ethical Committee of Erasmus Medical Center (number MEC-2018-120). The analysis was conducted using SPSS Statistics software (version 25; IBM Corp).

## Results

### Respondents’ Characteristics

Our sample consisted of 481 respondents. However, 18 respondents were excluded from the total for not filling out the questions regarding the dependent variable. The respondents in this sample had a mean age of 74 (SD 5.77) years. Almost all respondents (456/463, 98.5%) had prior experience with using the internet, but only 127 (26.6%) had ever used medical applications before. Further details on the respondent characteristics can be found in Table 1.

**Table 1:** Baseline characteristics of the study cohort (N=463).

Characteristics			Participants, n (%)
**Sex**			
	Male	231 (50.9)	
	Female	223 (49.1)	
**Age (years)**			
	65-74	270 (58.4)	
	75-84	164 (35.5)	
	≥85	28 (6.1)	
**Education level**			
	No or lower education	64 (13.9)	
	Intermediate education	232 (50.3)	
	Higher education	165 (35.8)	
**General health**			
	Poor	4 (0.9)	
	Fair	86 (18.6)	
	Good	215 (46.4)	
	Very good	108 (23.3)	
	Excellent	50 (10.8)	
**Prior experience with the internet**			
	Yes	456 (98.5)	
	No	7 (1.5)	
**Prior experience with mHealth**			
	Yes	127 (27.4)	
	No	336 (72.6)	
**Participation (data collection timepoint)**			
	February 2020	1 (0.2)	
	March 2020	108 (23.3)	
	April 2020	165 (35.6)	
	May 2020	188 (40.6)	
	June 2020	1 (0.2)	

### Cronbach Alpha and Correlation Analyses

Cronbach *α* scores, which show the internal consistency of the items within each acceptance factor, are shown in Table 2. The scores were well above the recommended limit of .70, indicating acceptable reliability [57].

The outcome of the correlation analysis is presented in Table 3. Perceived risk factors were found to be significantly and substantially correlated to one another. Therefore, the factors are not jointly included in the multivariate linear regression analysis. The control variables were also combined in a correlation analysis (see Multimedia Appendix 3). This analysis showed that the control variables were not substantially correlated to one another and could, therefore, be jointly included as control variables in the multivariate linear regression analysis.

**Table 2:** Cronbach *α* values for acceptance factors of the questionnaire.

Cluster (number of statements within a construct)	Cronbach *α*
Intention to use (n=3)	.959
Privacy risk (n=4)	.911
Performance risk (n=4)	.868
Legal concern (n=3)	.921
Trust (n=5)	.863

**Table 3:** Correlation analysis between the four risk factors.

Variable			Privacy risk		Performance risk		Legal concern		Trust
**Privacy risk**									
	*r*	1		0.510		0.719		–0.202	
	*P* value (2-tailed)	—^a^		<.001		<.001		<.001	
**Performance risk**									
	*r*	0.510		1		.576		–­0.323	
	*P* value (2-tailed)	<.001		—		<.001		<.001	
**Legal concern**									
	*r*	0.719		.576		1		–0.264	
	*P* value (2-tailed)	<.001		<.001		—		<.001	
**Trust**									
	*r*	–0.202		–0.323		–0.264		1	
	*P* value (2-tailed)	<.001		<.001		<.001		—	

^a^Not applicable.

### Univariate and Multivariate Analyses

The results of the univariate and multivariate regression analyses are summarized in Table 4. All factors were found to be significantly associated with intention to use mHealth. Privacy risk, performance risk, and legal concern were found to be negatively associated with intention to use, and trust was found to be positively associated with intention to use. In the multivariate regression analyses, we controlled for sex, age, education level, and health status. Privacy risk, performance risk, and legal concern continued to have a significantly negative coefficient. Performance risk had a negative coefficient of –0.266. This coefficient is large in comparison to the coefficients for privacy risk (–0.099) and legal concern (–0.124). Trust had the largest significant (positive) coefficient (0.350). The multivariate regression analyses (see Multimedia Appendix 3) also show that health status is significantly and negatively correlated to intention to use when considered a control variable for each of the four risk factors, whereas education was only significant in the model with trust. Age was significantly related to intention to use, except when being a control variable for legal concern. Sex was not found to be significant in any scenario.

**Table 4:** Results of univariate and multivariate linear regression analyses, and coefficients for perceived risk factors.

Variable	Univariate linear regression		Multivariate linear regression^a^			
	Standardized *β* coefficient (95% CI)	*P* value	Unstandardized coefficient B (95% CI)	Standardized *β* coefficient	*P* value	Adjusted R^2^
Privacy risk	–.124 (–0.224 to –0.034)	.008	–0.103 (–0.195 to –0.011)	–.100	.03	0.089
Performance risk	–.331 (–0.537 to –0.315)	<.001	–0.337 (–0.450 to –0.225)	–.266	<.001	0.147
Legal concern	–.167 (–0.284 to –0.085)	<.001	–0.136 (–0.235 to –0.038)	–.125	.007	0.093
Trust	.375 (0.464 to 0.735)	<.001	0.555 (0.422 to 0.687)	.352	<.001	0.202

^a^Adjusted for age, sex, education, and health status.

## Discussion

### Principal Results and Comparison With Prior Work

This study explored whether perceived risks influence the intention to use mHealth applications among independently living older adults. Our findings showed that privacy risk, performance risk, legal concern, and trust were significantly associated with participants’ intention to use mHealth applications. The perceived risks (ie, privacy risk, performance risk, legal concern) were negatively associated with intention to use, whereas *trust* was positively associated with intention to use.

Trust had the largest correlation coefficient and explained more than 20% of the variance in intention to use when the controls are added, indicating that it has a considerable positive effect on the behavioral intention to use mHealth. This finding indicates that older adults who perceive doctors accessible via mHealth applications as trustworthy and reliable have a higher intention to use these applications, as hypothesized in this study. These findings broadly confirm previous findings obtained for the general population in China and strengthen existing evidence that trust is an important determinant for the intention to use mHealth applications [35,51-53].

Performance risk was significantly and negatively associated with individuals’ intention to use mHealth. These findings confirm our hypothesis stating that independently living older adults who doubt whether mHealth applications can meet their health care needs have a lower intention to use these applications. This further confirms previously reported findings for the general population and may be more valid for older adults, as they are more likely to fear that technologies will replace health care professionals [35,45].

Legal concern was negatively and significantly associated with intention to use. This confirms our hypothesis, which was based on the argument that older adults who are more likely to worry about inappropriate law enforcement are less likely to have a behavioral intention to use mHealth. The significance of the relationship differs from previous research in which the relationship between legal concern and intention to use was not found to be significant [35]. This may be explained by the fact that Deng et al [35] addressed the general Chinese population, in the hospital context, wherein mHealth use is more limited and legal issues may be less of a concern to respondents because of specific health laws being in place in this context. In addition, discussions on legal aspects of mHealth applications received considerable attention in the Netherlands during the COVID-19 outbreak, especially about a “Corona App” [65,66]. This may have raised concerns among independently living older adults. Legal concerns regarding mHealth use appear to have received little attention in the scientific literature and form a relevant area for further research.

Privacy risk also was significantly and negatively associated with participants’ intention to use mHealth, albeit with a smaller coefficient. This finding confirms our hypothesis and is consistent with previous results reported by Deng et al [35] in the general Chinese population. Privacy risk is not directly linked to the functions of the mHealth app, as it involves the confidentiality of personal health information during the use of mHealth. Hence, compared with performance risk, privacy risk may likely exert less effect on the intention to use mHealth, which explains the smaller coefficient [35].

As the correlation analysis showed, the perceived risk factors are significantly and substantially correlated to one another. These results are not in accordance with the results from Deng et al [35], where a high discriminant validity was shown between the factors. This could be explained by the fact that Deng et al’s 2018 study included very few respondents of 65 years and above, and older adults are more prone to perceive mHealth risks than younger people [67,68]. Another related explanation might be that there are underlying shared determinants of the risks perceived by older adults.

A recent study from the Netherlands showed that the adoption rate of a COVID-19 tracing app was significantly lower for older adults than for young adults [69]. One of their hypotheses for this lower adoption rate was that older adults would feel insufficiently protected by a contract tracing app. Older adults felt insufficiently protected because of the different perceived risk factors as shown in our study, thereby leading to a lower adoption rate.

### Relationships Between Trust and Other Factors

Trust has been proposed to function as a mediator of five relationships between the three identified perceived risks (ie, privacy risk, performance risk, and legal concern) and the two TAM factors (perceived ease of use and perceived usefulness) with intention to Use [35]. Certainly, these hypotheses have intuitive appeal and, to some extent, theoretical support as well [35,37,38,41]. The empirical results of Deng et al [35] accept two of these five hypotheses and reject the other three.

The theoretical support for the relationship of these variables with trust clearly depends on the definition and operationalization of “trust.” Following the construct definition presented by Deng et al [35], the operationalization adopted in this study focuses on trust in the medical doctors that the mHealth applications connects the user with. Such trust in medical doctors is essentially different from trust in the technology itself, and the intuitive and theoretical arguments cannot be assumed to remain unaffected. In fact, there is literature to support that the technology acceptance factors are influenced by trust in “entities behind the system” [70], suggesting that trust is a determinant of perceived ease of use and perceived usefulness rather than a mediator of their effect on the intention to use.

As shown in Multimedia Appendix 3, the two TAM variables perceived usefulness and perceived ease of use are indeed significantly related to trust in our study. However, in view of the definition and the arguments above, further appropriately designed research on the nature and direction of these relationship is called for.

Similar reflections are in place regarding the relationships between trust and the three perceived risk factors. Multimedia Appendix 3 presents univariate and multivariate regression analyses with trust as the dependent variable for the three risk factors. It shows that performance risk, privacy risk, and legal concern all are negatively and significantly associated with trust. This finding contrasts with previous findings by Deng et al [35] who report that performance risk and privacy risk were significantly and negatively associated with trust, whereas legal concern was not significantly related to trust. This difference in findings might well be explained by a general difference in legal concern between the study populations, which differ in culture, age, and state. In view of the definition of trust, we call for caution to state whether any of the risks are a determinant of trust, and/or the other way around. These relationships deserve further appropriately designed research.

### Limitations

Our study has some limitations. First, this study was conducted during the COVID-19 pandemic, which complicated the data collection, as it became increasingly difficult to approach the targeted population. It became very difficult to recruit respondents who were unable or unwilling to fill out the web-based questionnaire. This may have resulted in a bias towards independently living older adults with better internet literacy. Second, we used a cross-sectional research design. Consequently, the causality of our findings cannot be claimed. Third, we noticed that some of the participants, especially those in the age group above 75 years, struggled to understand the use and utility of medical applications properly. To address this situation, the questionnaires and interviewers provided an additional explanation about medical applications. Finally, although the data are collected from a variety of contexts in the Netherlands, we cannot claim validity in other countries.

### Recommendations and Future Research

The main contribution of this study is to provide the first large-scale quantitative evidence of the validity and significance of the perceived risk factors determining intention to use mHealth applications among older adults in the Netherlands. In our study, we identified trust and performance risk as the most important factors that had a relation with the older adults’ intention to use mHealth services. We suggest involving older adults in the design and development of mHealth applications to ensure that the applications will be tailored to their needs and abilities. Moreover, we suggest involving medical specialists, geriatricians, and other experts in the development of these applications and making this explicit to potential users of the application to increase trust and diminish concerns about the performance of mHealth. Additionally, since privacy risks and legal concerns have a relation with the intention to use mHealth, we suggest that health care professionals, designers of mHealth applications, and the Dutch government use these findings to tailor their mHealth services and campaigns and address the concerns of older adults, to promote better adoption.

As we cannot confirm causality, we recommend studying perceived risk factors using controlled experiments rather than observational studies to confirm or disprove any potential causality of the relationships thus found. To further understand how the perceived risk factors explain behavioral intention to use, possibly via interaction with each other and the variables from models such as TAM, STAM, and UTAUT, qualitative studies are called for. Such qualitative studies can also enable effective solutions to eliminate barriers to medical application adoption. Furthermore, although the intention to use technology has been shown to predict actual usage [41], such experiments may research actual technology adoption, rather than the intention to use mHealth applications.

## Appendix

Multimedia Appendix 1 Constructs and statements used in this study.

Multimedia Appendix 2 Checklist for Reporting Results of Internet E-Surveys (CHERRIES).

Multimedia Appendix 3 Results of linear regression analyses and correlation analyses.

## Abbreviations

CHERRIES: Checklist for Reporting Results of Internet E-Surveys
mHealth: mobile health
TAM: technology acceptance model
UTAUT: unified theory of acceptance and use of technology
