# Supplementary material for: Relationship Between Perceived Risks of Using mHealth Applications and the Intention to Use Them Among Older Adults in the Netherlands: Cross-sectional Study
Source: JMIR Mhealth Uhealth. 2021 Aug 30;9(8):e26845. doi: 10.2196/26845 (PMC8438611; doi:10.2196/26845)
Supplement: Multimedia Appendix 1 [file mhealth_v9i8e26845_app1.docx]

Multimedia Appendix 1

**Table S1.** Constructs and statements used in this study.

| Construct | Scale items |
| --- | --- |
| Intention to Use | Assuming I have access to medical apps, I intend to use them. |
|  | Given that I have access to medical apps, I predict that I would use them. |
|  | Assuming I have access to medical apps, I intend to use them as much as possible. |
| Privacy risk | I am afraid that medical apps providers cannot guarantee the confidentiality of user information. |
|  | I am worried that my personal privacy information will be used for  other purposes if I use medical apps. |
|  | I am worried that when using medical apps, my personal information will be abused by cyber criminals. |
|  | Because of security issue, I am worried about personal  information leakage when I consult a doctor about sensitive health  problems on medical apps. |
| Performance  risk | I am worried that using medical apps cannot satisfy my health needs. |
|  | I am afraid that the health advice from medical apps cannot well address my health concerns. |
|  | Compared with the traditional offline office visits, I am concerned  about the quality of online health consultation. |
|  | I am worried that the services provided by medical apps may not match my expectations. |
| Legal concern | I am worried that my personal health-related information is not  protected by law when using medical apps. |
|  | I am worried that the behaviors of doctors and patients on medical apps lack special legal restrictions and may have adverse effects. |
|  | I am afraid that the rights and interests of users cannot be ensured  because of the lack of specific law enforcement on the medical apps. |
| Trust | Generally, I think doctors on medical apps are trustworthy. |
|  | Most of the doctors on the medical apps are health experts in their field, and I have no doubt about their profession. |
|  | Doctors in professional health care websites are verified, and I  think their credibility is guaranteed. |
|  | Health consultations with doctors online is reliable to solve my  health problems. |
|  | In general, I trust health advices or tips from doctors on the  internet. |
