# Supplementary material for: Relationship Between Perceived Risks of Using mHealth Applications and the Intention to Use Them Among Older Adults in the Netherlands: Cross-sectional Study
Source: JMIR Mhealth Uhealth. 2021 Aug 30;9(8):e26845. doi: 10.2196/26845 (PMC8438611; doi:10.2196/26845)
Supplement: Multimedia Appendix 3 [file mhealth_v9i8e26845_app3.docx]

Multimedia Appendix 3

To explore a possible intermediating effect of Trust on the relationship between each of perceived risk factors [35], we conducted univariate and multivariate regression analysis with trust as the dependent variable and the perceived risk factors as independent variables. The results are summarized in Table S1 below. In the univariate and the multivariate regression analysis, Privacy risk, Performance risk and Legal concern are significantly and negatively correlated with Trust. Performance risk has a negative coefficient of -0.317. Subsequent, Legal concern had a negative coefficient of -0.249 and Privacy risk of -0.184.

**Table S1.** Results of linear regression analyses and coefficients for perceived risk constructs, considering “trust” as the dependent variable

|  | Univariate linear regression | | Multivariate linear regression ^a^ | | | |
| --- | --- | --- | --- | --- | --- | --- |
|  | Standardized coefficient β (95% CI for B) | *P* value | Unstandardized coefficient B (95% CI for B) | Standardized coefficient β | *P* value | Adjusted R2 |
| **Privacy risk 🡪 Trust** | -0.202 (-0.191; -0.073) | <.001 | -0.123 (-0.183; -0.060) | -0.188 | <.001 | 0.044 |
| **Performance risk 🡪 Trust** | -0.323 (-0.329; -0.190) | <.001 | -0.254 (-0.327; -0.181) | -0.317 | <.001 | 0.104 |
| **Legal concern 🡪 Trust** | -0.264 (-0.243; -0.121) | <.001 | -0.177 (-0.240; -0.114) | -0.257 | <.001 | 0.076 |

^a^ Adjusted for age, sex, education, and health status

The univariate and multivariate linear regression shows that Perceived ease of use and Perceived usefulness have a significant positive correlation with Trust.

**Table S2.** Results of the linear regression analyses with the variables Perceived ease of use, Perceived usefulness and Trust

|  | Univariate linear regression | | Multivariate linear regression ^a^ | | |
| --- | --- | --- | --- | --- | --- |
|  | Standardized coefficient β (95% CI for B) | *P* value | Unstandardized coefficient B (95% CI for B) | Standardized coefficient β | *P* value |
| **Perceived ease of use 🡪 Trust** | 0.286 (0.151-0.284) | <.001 | 0.242 (0.169-0.315) | 0.311 | <.001 |
| **Perceived usefulness 🡪 Trust** | 0.345 (0.194-0.323) | <.001 | 0.266 (0.197-0.336) | 0.353 | <.001 |
| **Trust 🡪 Perceived ease of use** | 0.286 (0.260-0.490) | <.001 | 0.365 (0.255-0.476) | 0.285 | <.001 |
| **Trust 🡪 Perceived usefulness** | 0.345 (0.346-0.576) | <.001 | 0.429 (0.317-0.541) | 0.324 | <.001 |

^a^ Adjusted for age, sex, education, and health status

The correlation matrix shows that the control variables are not substantially correlated to one another and can, therefore, be jointly included as control variables in the multivariate linear regression analysis.

**Table S3.** Correlation matrix of control variables.

|  |  | Education | Sex | Health status |
| --- | --- | --- | --- | --- |
| Age | *r* | -.119 | .007 | -.071 |
|  | *P* value (2-tailed) | .011 | .883 | .130 |
| Education | *r* | - | -.140 | .034 |
|  | *P* value (2-tailed) | - | .003 | .470 |
| Sex | *r* | - | - | -.046 |
|  | *P* value (2-tailed) | - | - | .325 |

The univariate and multivariate linear regression analysis shows that age, higher education, and health status are significantly related to Intention to use mHealth. Age is negatively related to Intention to use. High education and good health status are positively related to Intention to use mHealth.

**Table S4.** Results of univariate and multivariate linear regression analyses with control variables.

|  |  | Univariate linear regression | | Multivariate linear regression with all the control variables together | | |
| --- | --- | --- | --- | --- | --- | --- |
|  |  | Standardized coefficient β | *P* value | Unstandardized coefficient B | Standardized coefficient β | *P* value |
| **Age** |  | -0.134 | .004 | -0.016 | -0.105 | .023 |
| **Sex** |  |  |  |  |  |  |
|  | Male (reference) | 0.056 | .230 | - | - | - |
|  | Female | -0.056 | .230 | -0.066 | -0.037 | .416 |
| **Education** |  |  |  |  |  |  |
|  | No/lower education (reference) | -0.115 | .014 | - | - |  |
|  | Intermediate education | -0.088 | .058 | 0.185 | 0.104 | .133 |
|  | Higher education | 0.175 | <.001 | 0.428 | 0.231 | .001 |
| **Health status** |  | 0.209 | <.001 | 0.196 | 0.200 | <.001 |

**Table S5.** Results of multivariate linear regression analysis with privacy risk and control variables.

|  |  | Multivariate linear regression | | |
| --- | --- | --- | --- | --- |
|  |  | Unstandardized coefficient B | Standardized coefficient β | *P* value |
| **Privacy risk** |  | -.103 | -.100 | .029 |
| **Age** |  | -.017 | -.106 | .021 |
| **Sex** |  |  |  |  |
|  | Male (reference) | - | - | - |
|  | Female | -.062 | -.035 | .445 |
| **Education** |  |  |  |  |
|  | No/lower education (reference) | - | - | - |
|  | Intermediate education | .171 | .097 | .162 |
|  | Higher education | .415 | .224 | .001 |
| **Health status** |  | .189 | .192 | <.001 |

**Table S6.** Results of the multivariate linear regression analysis with performance risk and control variables.

|  |  | Multivariate linear regression | | |
| --- | --- | --- | --- | --- |
|  |  | Unstandardized coefficient B | Standardized coefficient β | *P* value |
| **Performance risk** |  | -.337 | -.266 | <.001 |
| **Age** |  | -.012 | -.076 | .089 |
| **Sex** |  |  |  |  |
|  | Male (reference) | - | - | - |
|  | Female | -.059 | -.033 | .454 |
| **Education** |  |  |  |  |
|  | No/lower education (reference) | - | - | - |
|  | Intermediate education | .141 | .080 | .235 |
|  | Higher education | .336 | .181 | .008 |
| **Health status** |  | .159 | .162 | <.001 |

**Table S7.** Results of the multivariate linear regression analysis with legal concern and control variables.

|  |  | Multivariate linear regression | | |
| --- | --- | --- | --- | --- |
|  |  | Unstandardized coefficient B | Standardized coefficient β | *P* value |
| **Legal concern** |  | -.136 | -.125 | .007 |
| **Age** |  | -.015 | -.095 | .039 |
| **Sex** |  |  |  |  |
|  | Male (reference) | - | - | - |
|  | Female | -.045 | -.025 | .582 |
| **Education** |  |  |  |  |
|  | No/lower education (reference) | - | - | - |
|  | Intermediate education | .162 | .091 | .187 |
|  | Higher education | .412 | .223 | .002 |
| **Health status** |  | .182 | .185 | <.001 |

**Table S8.** Results of multivariate linear regression analysis with trust and control variables.

|  |  | Multivariate linear regression | | |
| --- | --- | --- | --- | --- |
|  |  | Unstandardized coefficient B | Standardized coefficient β | *P* value |
| **Trust** |  | .555 | .352 | <.001 |
| **Age** |  | -.017 | -.110 | .011 |
| **Sex** |  |  |  |  |
|  | Male (reference) |  |  |  |
|  | Female | -.025 | -.014 | .741 |
| **Education** |  |  |  |  |
|  | No/lower education (reference) |  |  |  |
|  | Intermediate education | .266 | .151 | .021 |
|  | Higher education | .451 | .244 | <.001 |
| **Health status** |  | .163 | .167 | <.001 |
